# Prevalence and associated factors of pre-eclampsia among pregnant women attending anti-natal care at Mettu Karl referal hospital, Ethiopia: cross-sectional study

**DOI:** 10.1186/s40885-019-0120-1

**Published:** 2019-07-01

**Authors:** Alemayehu Sayih Belay, Tofik Wudad

**Affiliations:** Department of Nursing, College of Health Sciences, Mizan Tepi University, P.O. Box 260, Mizan Teferi, Ethiopia

**Keywords:** Factors, Prevalence, Preeclampsia, Pregnant women, Anti-natal care

## Abstract

**Background:**

Preeclampsia is leading causes of maternal and perinatal morbidity and mortality worldwide and it is a hypertensive disorder which usually occurs after 20 weeks of gestation. In Ethiopia, according to Ethiopian National Emergency Obstetric and Newborn Care about 10% of all maternal mortality (direct and indirect) were due to preeclampsia. Despite this condition has adverse effects on the maternal and child health, its prevalence is still significant especially in developing countries including Ethiopia.

**Objectives:**

The aim of the study is to assess the prevalence and associated factors of preeclampsia among pregnant women attending antenatal care at Mettu Karl referral hospital.

**Method:**

The study was conducted at Mettu Karl referral hospital using institutional based cross sectional study design among women whose age was greater or equal to eighteen from March to April 2018. Data were collected from 129 participants by face to face interview technique using structured and pretested questionnaire. Logistic regression analysis was used to identify the factors associated with preeclampsia development.

**Result:**

A total of 129 participants were enrolled in the study with the mean age of 25.87 [SD ± 4.757]. Prevalence of preeclampsia among the current pregnant women who attend ANC in Mettu Karl Hospital were 16 (12.4%) with 95% CI (7, 18). Predictor variables like respondents age (AOR = .009, 95% CI = [.000, .317]), current multiple pregnancy (AOR = .071, 95% CI = [.007, .773]) and history of diabetes mellitus (AOR = .058, 95% CI = [.007–.465]) were significantly associated with the current preeclampsia.

**Conclusion:**

The finding of this study showed that a considerable proportion of women had preeclampsia. Health seeking behavior towards pregnant women’s should be encouraged for both urban and rural residents, which provide a chance to diagnose preeclampsia as early as possible and to prevent the coming complication towards preeclampsia.

## Introduction

Preeclampsia is the leading causes of maternal and perinatal morbidity and mortality worldwide and it is a hypertensive disorder which usually occurs after 20 weeks of gestation. It is a rapidly progressive condition characterized by elevated blood pressure and protein in the urine [1, 2]. It is a cause of severe morbidity, long term disability and death among both mothers and their babies. The risk of maternal death is much more common in settings in which prenatal and intra partum care is not routinely available to pregnant women [3].

Every day in 2015, about 830 women died due to complications of pregnancy and childbirth. Almost all of these deaths occurred in low-resource settings, and most could have been prevented [4]. Pregnancy induced hypertensions like preeclampsia is one of the primary causes of death [5, 6]. Of the 830 daily maternal deaths, 550 occurred in Sub-Saharan Africa and 180 in Southern Asia, compared to 5 in developed countries. The risk of a woman in a developing country dying from a maternal-related causes during her lifetime is about 33 times higher compared to a woman living in a developed country [7].

The prevalence of preeclampsia in developing countries ranges from 1.8 to 16.7% [8]. For instance, the prevalence of preeclampsia occurs in 10% of pregnancies in African women, which is significantly higher than the global average of approximately 2% [9].

In Ethiopia, the estimate of the maternal mortality ratio for the 7-year period preceding the 2016 Ethiopian demographic health survey (EDHS) is 412 deaths per 100,000 live births; that is, for every 1000 births in Ethiopia, there are about 4 maternal deaths [5].

A 5 year retrospective review in Ambo Hospital found that 12.3% maternal mortality occurred due to hypertension disorder of pregnancy [10]. Moreover, according to Ethiopian National Emergency Obstetric and Newborn Care (EMONC) 16% of direct maternal mortality and 10% of all maternal mortality (direct and indirect) was due to preeclampsia/eclampsia [11].

Despite this condition has adverse effects on the maternal and child health, [12] its prevalence is still significant especially in developing countries including Ethiopia. In general, preeclampsia remains a major problem both in maternal and infants morbidity and mortality. But the risk factors for preeclampsia have not been well documented in Ethiopia. Hence, this study can assess the prevalence and its associated factors of preeclampsia among pregnant women in Mettu Karl Referral hospital, South West Ethiopia.

## Methodology

### Study area

The study was conducted at Mettu Karl referral hospital found in **Metu** (also Mettu) which is a market town and separate woreda in South West Ethiopia, Oromia region, Ilu-Ababor Zone. It is located 600 km from the capital city of Ethiopia, Addis Ababa. Mettu, located in the Illubabor Zone of the Oromia Region along the Sor River, this town has a latitude and longitude of 8°18′N 35°35′E and an altitude of 1605 m. This hospital is being given different services for clients referred from health centers around Illubabor. It also gives service for mothers who suffer from fistula at its fistula center. Generally the hospital can give service for around 1.4 million clients. Regarding the delivery information, the average annual number of delivering mothers is estimated to be 3602.

### Study design, period and population

Institutional based cross sectional study design was conducted among women whose age was greater or equal to eighteen from March to April 2018. Selected pregnant women who gave at least one child birth and those who were above 20 weeks of gestation for current pregnancy were included.

### Sample size determination

The sample size was determined using single population proportion formula using the following assumptions; the magnitude of preeclampsia, which is 8.4% in Dessie referral hospital [13], confidence interval 95%, the *margin of error* d = 0.05, Za/2 = 1.96 and with 10% non-respondent rate the final sample size was 129.

### Sampling procedure and data collection instrument

First, we obtained the annual report of the pregnant mothers with gestational age of greater than 20 weeks in ANC then we divide by 12 to get the flow of pregnant mothers per month. Then finally we found about 139 pregnant mothers who come for ANC follow up. Then the total sample size required was collected consecutively within the given period.

The questionnaire is adopted and modified from reviewing different literatures and scientific facts [1, 13–16]. Data were collected by face to face interview technique using structured and pretested questionnaire. Medical records were also reviewed for some clinical and laboratory results, including proteinuria and blood pressure measurement.

### Data quality control

A questionnaire was first prepared in English and then translated to national language (Amharic) and was translated back to English by another individual in order to check and maintain its consistency.

After necessary modifications and correction was made and ensured its reliability by the pre-test, the actual data were collected by four midwife nurses. To maintain the quality of the data the 3 days of training was given for the data collectors. Questionnaires which were collected were checked for its completeness and consistency of the filled questionnaires on a daily basis. Blood pressure was taken under standard operating procedure by two data collectors for each participant, to keep its reliability of measurement and to correctly diagnose preeclampsia. Current multiple pregnancy was also confirmed by both the physical examination and ultrasound evaluation. The medical registration numbers of the participants who were involved in the study were recorded on a separate sheet to avoid repeated recruitment of the study participants who come for the next visit.

In addition, the data were thoroughly cleaned and carefully entered into computer for beginning of analysis.

### Operational definition

**Gestational age**: is calculated from the last normal menstrual period (LNMP) and for those women who didn’t recall their last menstrual period, fundal height and/or ultrasound result was used.

**Gravidity**: the total number of pregnancies, including abortion, ectopic pregnancy and any other pregnancies documented on the chart.

**Parity:** the number of deliveries after 28 weeks of gestation including IUFD and still birth documented in the chart.

**Pre-eclampsia**: denotes for women with blood pressure of ≥140 mmHg systolic or ≥ 90 mmHg diastolic on two separate readings taken at least four to six hours apart after 20 weeks gestation in an individual with previously normal blood pressure and proteinuria in pregnancy [1, 17].

**Proteinuria**: is assessed using the urine dipstick method. Those women levels of + 1 and above are classified as having proteinuria.

### Data processing and analysis

Data was entered using epi-data manager and exported, cleaned and analyzed using statistical package for social science (SPSS) version 21.

The multicollinarity between the variables were checked and there were no interaction among independent variables. We also checked the linear relationship between the continuous variable age and its logit transformation and finally we found that there was no linear relationship. As a result the independent variable age was categorized. Each independent/ predictor variables and outcome variable was investigated using bivariate logistic regression model. The independent variables that were statistically significant with *p value < 0.05* and 95% CI at bivariate analysis was included in multivariate logistic regression model using enter method to control for potential confounder variables. Finally, variables (age, current multiple pregnancy and participant history of diabetic mellitus) with *p value < 0.05* were taken as strong predictor variables of preeclampsia.

### Ethical consideration

Approval letter was obtained from Mizan-Tepi University, college of medicine and health sciences. The necessary information regarding to the importance of the study was addressed for each participant. Written consent was taken from each participant and their confidentiality and privacy was maintained.

## Result

### Socio-demographic characteristics of the study participants

A total of 129 participants who come for ANC follow up in Mettu Karl hospital were enrolled in the study with the 100% response rate. The mean age of the participants were 25.87 [SD ± 4.757]. About 47 (36.4%) and 37 (28.7%) of the participants were in the age range of 25–29 and 30–34 respectively.

Majority of the participants, 113 (87.6%) were married and most of them, 46 (35.7%) were Protestant by religion. More than half of the participants, 74 (57.4%) were housewife by their occupational status, whereas 18 (14.0%) of the respondents ever had more than one partner (Table 1).

**Table 1:** Socio demographic characteristics of the respondents (*n* = 129), Mettu Karl Referral Hospital, South West Ethiopia, 2018

Variables	Frequency	%
Place of residence		
Rural	53	41.1
Urban	76	58.9
Age of respondents		
< =24	30	23.3
25–29	47	36.4
30–34	37	28.7
> =35	15	11.6
Mean ± SD	25.87 ± 4.757	
Marital status		
Married	113	87.6
Single	5	3.9
Divorced	3	2.3
Widowed	4	3.1
Separated	4	3.1
Religion		
Muslim	37	28.7
Orthodox	45	34.9
Protestant	46	35.7
Catholic	1	.8
Ethnicity		
Oromo	97	75.2
Amhara	21	16.3
Tigre	7	5.4
Other^a^	4	3.1
Respondent‘s educational status		
Unable to write and read	13	10.1
Able to read and write	29	22.5
Primary	33	25.6
Secondary	23	17.8
Certificate and above	31	24.0
Respondent‘s occupational status		
House wife	74	57.4
Governmental Employee	29	22.5
Merchant	24	18.6
Student	2	1.6
Husband occupational status		
Farmer	40	31.0
Governmental employee	38	29.5
NGO employee	7	5.4
Merchant	35	27.1
Husband educational status		
Unable to write and read	27	20.9
Able to read and write	29	22.5
Primary	24	18.6
Secondary	25	19.4
Certificate and above	24	18.6
Ever had more than one partner		
Yes	18	14.0
No	111	86.0

^a^indicates that Gurage and Sheka

### Obstetrical characteristics of the respondents

Most of the participants age at first pregnancy, 118 (91.5%) were > =18. Of the total number of the current pregnancy, 11 (8.5%) were unintended pregnancy. About 87 (67.4%) of the participants ever had a history of ANC follow up during previous pregnancy, whereas 13 (10.1%) of the total participant had a history of abortion (Table 2).

**Table 2:** Obstetrical characteristic of respondents, Mettu Karl Referral Hospital, South West Ethiopia, 2018

Variables	Frequency	%
Age at first pregnancy		
< 18	11	8.5
> =18	118	91.5
Status of current pregnancy		
Intended	118	91.5
Unintended	11	8.5
Presence of current multiple pregnancy		
Yes	15	11.6
No	114	88.4
Gravidity		
< =2	43	33.3
> 2	86	66.7
Parity		
< 2	19	14.7
2–3	48	37.2
> =4	62	48.1
Ever had history of ANC follow up during previous pregnancy		
Yes	87	67.4
No	42	32.6
History of abortion		
Yes	13	10.1
No	116	89.9

### Prevalence of preeclampsia and clinical characteristics of the respondents

Prevalence of preeclampsia among the current pregnant women who attend ANC at Mettu Karl hospital were 16 (12.4%) with 95% CI [7, 18]. About 124 (96.1%) of the participants were ever heard about dander sign. Among the total participants, 13 (10.1%) of them had a family history of chronic hypertension, whereas 7 (5.4%) of the total participants had a family history of kidney diseases (Table 3).

**Table 3:** Prevalence of preeclampsia and clinical characteristics of the respondents, Mettu Karl Referral Hospital, South West Ethiopia, 2018

Variables	Frequency	%
Mother with preeclampsia		
No	113	87.6
Yes	16	12.4
Woman’s BP measurement at time of diagnosis		
< 140/90	108	83.7
140/90–160/110	16	12.4
> =160/110	5	3.9
Ever heard about danger sign		
Yes	124	96.1
No	5	3.9
Utilization of health facility for other health problem during current pregnancy		
Yes	60	46.5
No	69	53.5
History of chronic hypertension		
Yes	6	4.7
No	123	95.3
Family history of chronic hypertension		
Yes	13	10.1
No	116	89.9
Family history of pregnancy induced hypertension		
Yes	3	2.3
No	126	97.7
Family history of diabetes mellitus		
Yes	7	5.4
No	122	94.6
History of kidney disease		
Yes	42	32.6
No	87	67.4
Family history of kidney diseases		
Yes	7	5.4
No	122	94.6

### Factors associated with preeclampsia

On bivariate analysis, different independent variables like; age, residence, ever had more than one partner, current multiple pregnancy, family history of diabetes mellitus, the participant history of diabetes mellitus, the participant history of kidney disease, ever had a history of ANC follow up during previous pregnancy and parity were significantly associated with mothers with preeclampsia. But after adjusting for the possible confounding factors using multivariate analysis, variables like age, current multiple pregnancy and participant history of diabetic mellitus were become good independent predictor variables for preeclampsia.

Respondents whose age were < =24 were less likely to be preeclamptic women than those whose age were > =35 (AOR = **.009**, 95% CI = [**.000, .317**]). Participants who had no current multiple pregnancy were less likely to be preeclamptic women than those women with current multiple pregnancy (AOR = **.071**, 95% CI = [**.007, .773**]). Participant with no history of diabetes mellitus were less likely to be preeclamptic than those with a history of diabetes mellitus (AOR = **.058**, 95% CI = [**.007–.465**]) (Table 4).

**Table 4:** Factors associated with preeclampsia among women attending antenatal clinic at Mettu Karl referral Hospital, 2018

Variables	Mother with preeclampsia		COR (95% C. I)	AOR (95% C.I)	
	No (*n*)	Yes (*n*)			*P*-values
Age					
< =24	29	1	.039(.004–.369)	.009(.000–.317)*	.010
25–29	42	5	.136(.034–.538)	.362(.030–4.332)	.423
30–34	34	3	.101(.021–.478)	.136(.009–2.020)	.147
> =35	8	7	1.00^a^	1.00^a^	
Residence					
Rural	40	13	7.908 (2.127–29.405)	5.043(.670–37.96)	.116
Urban	73	3	1.00^a^	1.00^a^	
Ever had more than one partner					
Yes	11	7	7.212 (2.245–23.174)	1.57(.149–16.51)	.707
No	102	9	1.00^a^	1.00^a^	
Current multiple pregnancy					
Yes	8	7	1.00^a^	1.00^a^	
No	105	9	.098(.029–.332)	.071(.007–.773)*	.030
Family history of diabetic mellitus					
Yes	4	3	6.288 (1.265–31.26)	1.63(.091–29.35)	.741
No	109	13	1.00^a^	1.00^a^	
Participant history of diabetes mellitus					
Yes	13	11	1.00^a^	1.00^a^	
No	100	5	.059(.018–.197)	.058(.007–.465)*	.007
History of kidney disease					
Yes	32	10	4.219 (1.416–12.57)	1.345(.183–9.89)	.771
No	81	6	1.00^a^	1.00^a^	
Ever had history of ANC follow up during previous pregnancy					
Yes	81	6	.237(.080–.706)	.260(.021–3.151)	.290
No	32	10	1.00^a^	1.00^a^	
Parity					
< 2	16	3	.869(.216–3.507)	2.55(.245–26.56)	.434
2–3	46	2	.202(.042–.958)	.204(.017–2.407)	.207
> =4	51	11	1.00^a^	1.00^a^	

*Adjusted for all significant variables p < 0.05

^*a*^ *= Reference Category*

## Discussion

This institutional based cross sectional study was conducted in Mettu Karl referral hospital to identify the prevalence of preeclampsia and its associated factors.

In addition to this, the finding of this study will have a significant role towards overcoming the problems associated with preeclampsia and which in turn helps to decrease the maternal morbidity and mortality associated with preeclampsia.

Hence, in this study, we found that the prevalence of preeclampsia among the current pregnant women who attend ANC at Mettu Karl hospital were 16(12.4%).

This study finding was found to be high as compared with the studies conducted in different areas where it was found to be 3% in Norway [18], 2.31% in German in 2006 [19], 9.5% in Mustafa hospital of Ilam in the west of Iran [20] and 0.17% in Shiraz Southern Iran [21] and 4.8% in Brazzaville Teaching Hospital in the demographic republic of Congo DRC [22]. In comparison with the other studies conducted in different areas of the world, it was also found to be high as compared with the studies conducted in different parts of Ethiopia, where 8.4% in Dessie referral hospital [13] and 2.23% in Dilla [16]. This discrepancy might be due to the fact that there is a difference in study setting, socio-economical difference, methodological difference and a difference in time duration. For instance, in Norway the study was conducted for 40 years secular trends in epidemiology of preeclampsia and in DRC a prospective and transversal study was implemented where it was not conducted in this study.

In contrast to the other study, this study finding was found to be lower compared with the studies conducted in different areas where 16% in Nigeria [23] and 51.9% in Jimma University Specialized Hospital [24]. Theses difference might be attributed to the difference in socio-demographic and methodological difference. For example, large numbers of study participants where 1257 participants were included in the study in Nigeria but 129 study participants were included in this study. In addition to this, in Jimma the prevalence revealed that it was among those participants with PIH excluding participants with no PIH whereas in this study the prevalence showed among all participants.

Different independent predictor variables for preeclampsia were identified in this study.

In this study, maternal age was found to be significantly associated with preeclampsia. Respondents whose age were less than or equal to 24 were less likely to be preeclamptic women than those whose age were greater than or equal to 35. This is congruent with the study conducted in Germany [19], in Pakistan [25], in Tehran, Iran [26] and in Ethiopia, Desse [13]. This could be explained as the age increases, arteries will be clogged, which could lead to serious repercussions such as strokes or heart attacks. As the age increased, there will be poor diet or lack of exercise and ability of the body to process dietary salt, which in turn causes reduced elasticity of the blood vessels and finally causes the women to be hypertensive [27].

Multiple gestation was also significantly associated with preeclampsia. In this study, respondents who had no current multiple pregnancies were less likely to be preeclamptic than those women with current multiple pregnancy. This study finding was consistent with the studies conducted in Egypt [28], and in Ireland [29]. This might be due to the fact that, preeclampsia is associated with the presence of soluble substance which is a circulating antiangiogenic molecule of placental origin, plays a central role in preeclampsia by antagonizing placental growth factor (PIGF) and vascular endothelial growth factor signaling in the maternal vasculature [30].

History of diabetes mellitus was also another independent predictor variable for preeclampsia. Women with no history of diabetes mellitus were less likely to be preeclamptic than those with a history of diabetes mellitus. This finding is in line with the studies conducted in Sweden [31], in Ireland [29] and in Germany [32]. This might be explained by that diabetes is a disease in which the blood glucose, or blood sugar, levels are too high which will cause narrowing of blood vessels and interfere with the normal physiological response during pregnancy.

## Conclusion

The prevalence of preeclampsia in this study was higher than other similar studies. Predictor variables like; the age of participant, having multiple pregnancies and having a history of diabetes were factors associated with preeclampsia. Therefore, health seeking behavior towards pregnant women’s should be encouraged for both urban and rural residents, which provide a chance to diagnose preeclampsia as early as possible and to prevent the coming complication towards preeclampsia.

### Limitation of study

Even though this study contributes as an input for the policy makers towards the decrement of the maternal morbidity and mortality, it has its own limitations. First, since cross-sectional study design was implemented, it can’t establish cause effect relationship between the predictor variables and dependent variables. Second, recall bias might be expected for the last menstrual period. Third, because of resource limitation, we only incorporated one hospital as study area. Finally, since the study was institutional based study, the result will not be generalizable to the general population of the catchment area.

## Abbreviation

ANC: Ante natal care
BP: Blood pressure
DBP: Diastolic blood pressure
EDHS: Ethiopian demographic health survey
EMONC: Emergency obstetric and newborn care
LNMP: Last normal menstrual period
mmHg: Millimeter of mercury
PIH: Pregnancy induced hypertension
SBP: Systolic blood pressure
